# Effect of Exercise or Combined Exercise and Diet Intervention on Sleep and Fatigue in Kidney Transplant Recipients

**DOI:** 10.1016/j.ekir.2025.04.008

**Published:** 2025-04-10

**Authors:** Tim J. Knobbe, Daan Kremer, Monica L. van der Meulen, Frederike J. Bemelman, Stefan P. Berger, Gerjan Navis, Stephan J.L. Bakker, Eva Corpeleijn, ACTx Collaborators, Tim J. Knobbe, Daan Kremer, Dorien M. Zelle, Gerald Klaassen, Desie Dijkema, Iris M.Y. van Vliet, Paul B. Leurs, Frederike J. Bemelman, Maarten H.L. Christiaans, Stefan P. Berger, Gerjan Navis, Stephan J.L. Bakker, Eva Corpeleijn

**Affiliations:** 1 Department of Internal Medicine, Division of Nephrology, University of Groningen, University Medical Center Groningen, Groningen, The Netherlands; 2 Department of Internal Medicine, Division of Nephrology, University of Amsterdam, Amsterdam University Medical Center, Amsterdam, The Netherlands; 3 Department of Epidemiology, University of Groningen, University Medical Center Groningen, Groningen, The Netherlands

**Keywords:** diet, exercise, fatigue, lifestyle, physical activity, sleep

## Abstract

**Introduction:**

Physical functioning and diet may be promising targets to improve sleep and reduce fatigue among kidney transplant recipients (KTRs). We investigated whether a lifestyle intervention consisting of exercise or exercise combined with diet improve self-reported sleep and fatigue among KTRs.

**Methods:**

A predefined analysis of the Dutch multicenter randomized controlled Active Care after Transplantation (ACT) study (NCT01047410) was performed. Participants were allocated to control, exercise or exercise plus diet. The exercise group received 3 months supervised exercise with 15 months lifestyle coaching. This was supplemented with 15 months dietary counseling for the exercise plus diet group. Sleep and fatigue were assessed using the Kidney Disease Quality of Life-Short Form (KDQOL-SF) and the checklist of individual strength 20 (CIS20).

**Results:**

We included 146 KTRs (36% female, mean age: 54 ± 1 yr); 44 received usual care, 57 exercise intervention, and 45 exercise plus diet intervention. Mixed model analyses showed comparable sleep and fatigue trajectories across groups. At 15 months, no effects of the exercise intervention were observed for sleep (−2.5 arbitrary units [AU], 95% confidence interval [CI]: −9.1 to 4.2) and fatigue (−1.4 AU, 95% CI: −7.0 to 4.2). Similarly, at 15 months, no effects of the exercise plus diet intervention were observed for sleep (−1.4 AU, 95% CI: −8.4 to 5.6) and fatigue (−0.2 AU, 95% CI: −6.2 to 5.8). Fatigue improved compared with baseline at all time points (*P* ≤ 0.001). Of those with severe fatigue, 96% were able to follow the study protocol.

**Conclusion:**

Although patient-reported fatigue improves in the first year posttransplantation, (severe) fatigue remains highly prevalent and persisting among KTRs. Participation in a lifestyle rehabilitation program is feasible for KTRs with severe fatigue, but it neither improves nor worsens sleep or fatigue.

## Graphical abstract

**Figure:**
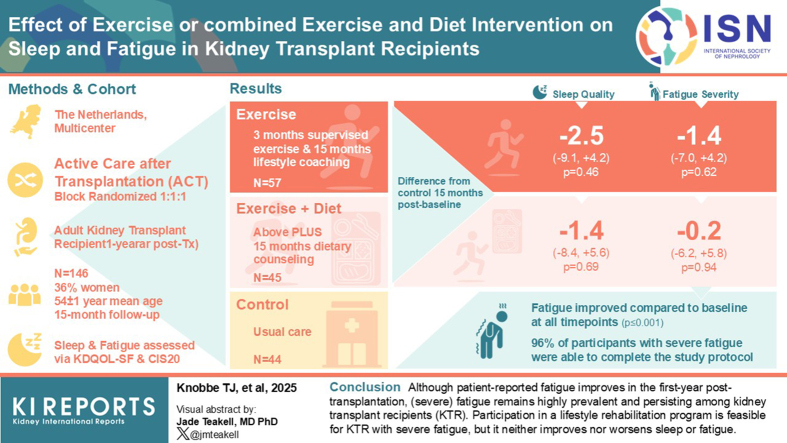

Kidney transplantation is the preferred treatment for kidney failure. However, even after successful transplantation, the health-related quality of life (HRQoL) of KTRs remains lower compared with the general population.^1^ Poor sleep and fatigue, both highly prevalent among KTRs, are considered important causes of this limited HRQoL.^2^^,^^3^ Intervention studies in other populations showed that improving physical functioning and diet can enhance sleep quality and reduce fatigue.^4^, ^5^, ^6^, ^7^, ^8^, ^9^ Findings in observational studies among KTRs align with these findings, showing associations of physical activity, protein intake, protein-energy wasting, body mass index, and other lifestyle factors such as smoking behavior with worse sleep quality or fatigue.^2^^,^^10^, ^11^, ^12^ However, evidence from intervention studies in KTRs is lacking.

Recently, the ACT study demonstrated that a 3-month supervised exercise intervention, combined with 15 months of lifestyle counseling, effectively improved muscle strength and cardiorespiratory fitness in KTRs.^13^ In addition, the intervention enhanced HRQoL-domain physical functioning at the end of the supervised exercise therapy. Given its proven effectiveness in improving physical fitness, this study was well-suited for investigating the impact of physical fitness on sleep and fatigue. The study also examined the exercise intervention combined with a 15-month diet intervention, where improvements in physical fitness and HRQoL did not reach statistical significance. However, its potential effects on sleep and fatigue remain to be investigated.

Therefore, we aimed to investigate whether the assessed 2 lifestyle interventions in this study, an exercise intervention or an exercise combined with diet intervention, improved self-reported sleep and fatigue among KTRs by performing a predefined *post hoc* analysis of this multicenter randomized controlled ACT study.

## Methods

This study is described following the CONSORT checklist when reporting of multiarm parallel-group randomized trials (Supplementary Table S1).^14^

### Study Design and Participants

Data from the parallel-group multicenter randomized controlled trial ACT study (NCT01047410) were used. A detailed description of the study protocol, study execution, and results of the primary and secondary aims have been published elsewhere.^13^ Trial inclusion ran from October 2010 to November 2016. The trial was approved by the University Medical Center Groningen Medical Ethic committee (NL49084.042.14; Groningen, The Netherlands). All clinical and research activities being reported are consistent with the Principles of the Declaration of Istanbul as outlined in the “Declaration of Istanbul on Organ Trafficking and Transplant Tourism,” and adhere to the Declaration of Helsinki.

Participants were included across 6 Dutch hospitals. KTRs up to 1-year posttransplant were eligible for participation. Other inclusion criteria were aged > 18 years and informed written informed consent. In the current study, we included only KTRs with data at baseline and at least 1 follow-up measurement of sleep or fatigue. Exclusion criteria included multiorgan transplantation, physical or clinical limitations that made safe participation impossible, psychopathology or serious cognitive impairment, and pregnancy.

### Randomization

**Figure 1:**
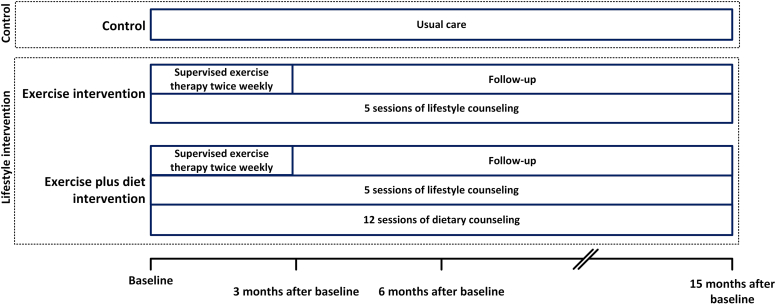
Study design. Participants were randomized 1:1:1 to control group, exercise intervention group, or exercise plus diet intervention group.

Participants were randomized 1:1:1 by block randomization in groups of 6 to 9 for rehabilitation centers and in groups of 3 for physiotherapy practices as the control (usual care), exercise intervention, or exercise plus diet intervention (Figure 1), by a data management company. Participants and investigators were unblinded; however, questionnaire data were collected by the independent data management organization.

### Control Group

The control group received care as usual in line with local treatment protocols. Patients were advised to have ≥ 30 minutes moderate physical activity per day. Standard nutritional care provided by a renal dietician, included 2 consultations, and focused on optimal nutrition, body weight, and muscle status, following the Dutch Dietary Guidelines for KTRs.

### Exercise Intervention

The exercise intervention included 2 phases as follows: an active phase consisting of an intensive 3-month supervised exercise program, followed by a less active phase in which participants were encouraged to maintain their active lifestyle. The supervised exercise program consisted of a 1.5-hour training session twice weekly for 3 months. Each session consisted of 30 minutes of dynamic resistance training of all major muscle groups, 30 minutes aerobic training, 30 minutes break, and 30 minutes supervised sports activity. The sessions were guided by trained and specialized physiotherapists. The intensity was tailored to individual baseline measurements and were in line with the American College of Sports Medicine exercise guidelines. The supervised exercise therapy was combined with lifestyle counseling for 15 months (i.e., until 1 year after the end of the supervised exercise therapy), in which the patients were encouraged to participate in sports and increase daily physical activity. The self-determination theory, stages of change model, and motivational interviewing were applied. In addition, patients received tools and materials, including a step counter to stimulate an active lifestyle. An extensive description of the exercise intervention has been published elsewhere.^13^^,^^15^

### Exercise Plus Diet Intervention

Participants received the same exercise intervention, combined with 15 months of dietary counseling, provided by a renal dietician in 12 sessions. The intervention was based on the Dutch Dietary Guidelines (for the general population, KTRs, preventing diabetes, and preventing cardiovascular disease), and personal goals were determined. Food intake was evaluated with 3-day food diaries. In case of (or risk of) malnutrition, energy-rich and protein-rich diets were advised. An extensive description of the exercise plus diet intervention has been published elsewhere.^13^^,^^15^

### Outcomes

Self-reported sleep was assessed using KDQOL-SF, validated in the kidney transplant population,^16^ sleep domain, consisting of 4 items, in which various aspects of sleep are assessed, including overall sleep quality, frequency of nighttime awakenings with difficulty returning to sleep, adequacy of sleep, and difficulty staying awake during the day.^16^^,^^17^ The questionnaire has a recall of 4 weeks and scores can range from 0 to 100, with a higher score reflecting better sleep. Self-reported fatigue was measured using the CIS20 severity domain, which consists of 8 items and questions about the intensity of fatigue in the past 2 weeks.^18^^,^^19^ It includes items that assess how tired, exhausted, fit, active, weak, lively, and rested the person feels, as well as whether they physically feel to be in a bad condition. Scores can range from 8 to 56, with a higher score reflecting more fatigue. A score ≥ 35 AU is indicative of severe fatigue. This questionnaire has been extensively validated in other populations with chronic diseases,^20^ and has been used in the kidney transplant population.^2^^,^^21^, ^22^, ^23^, ^24^ Questionnaires were collected by an independent data management company. They were sent to participants in paper form via regular mail; upon completion, the participants returned the questionnaires by mail.

### Covariables

Baseline demographic and clinical data were retrieved from medical records; educational level and smoking behavior were obtained by questionnaires. Sex was defined as sex assigned at birth. Laboratory measurements were performed by standard laboratory methods. Estimated glomerular filtration rate was calculated using the 2021 creatinine-based Chronic Kidney Disease-Epidemiology Collaboration equation.^25^ The study was not permitted to collect data on ethnicity by the Medical Ethics Committee.

### Statistical Analysis

Normally distributed data were presented as mean ± SD, nonnormally data as median [interquartile range] and categorical data as number (valid %). Between-group differences were assessed using 1-way analysis of variance test, Kruskal Wallis test or chi-square test, depending on the data distribution.

The same analytical approach as in the primary paper was applied.^13^ The primary analyses applied the intention-to-treat approach. General linear mixed model analyses were performed. The fixed factors were the allocation groups and time, which was added as a categorical variable, to allow a nonlinear trajectory, given the 2 study phases. The study center was added as a random factor, to account for differences between centers in the posttransplant care, inclusion of participants, and execution of the study protocol. An unstructured covariance structure modeled within-patient errors, and the Satterthwaite approximation calculated degrees of freedom. The dependent variable was the difference compared with baseline. Potential interactions by sex, age, time since transplantation, history of dialysis, donor type, educational level, systolic blood pressure, active smoker, severe fatigue at baseline, or baseline sleep quality (median value, because of lack of an accepted cutoff) on the treatment effect on sleep quality or fatigue severity were assessed by including these variables and their interaction terms as fixed factors in the model. For significant interactions, subgroup analyses were performed.

To assess whether sleep or fatigue improved compared with baseline during the study period, we repeated the mixed model analyses while combining all 3 groups, because no significant differences between groups were observed at each time point.

In per-protocol analyses, participants who did not adhere to or did not complete the intervention (e.g., by discontinuing or death) were excluded. Attendance at scheduled sessions with exercise and diet providers was used to assess adherence, with less than 75% attendance defined as nonadherence.

A 2-tailed alpha < 0.05 was deemed statistically significant. Data analyses were conducted using R (version 4.0.5; R Foundation for Statistical Computing, Vienna, Austria) and SPSS Statistics (version 28; IBM Corp, Armonk, NY), with figures created in GraphPad Prism (version 9; GraphPad, San Diego, CA).

## Results

**Figure 2:**
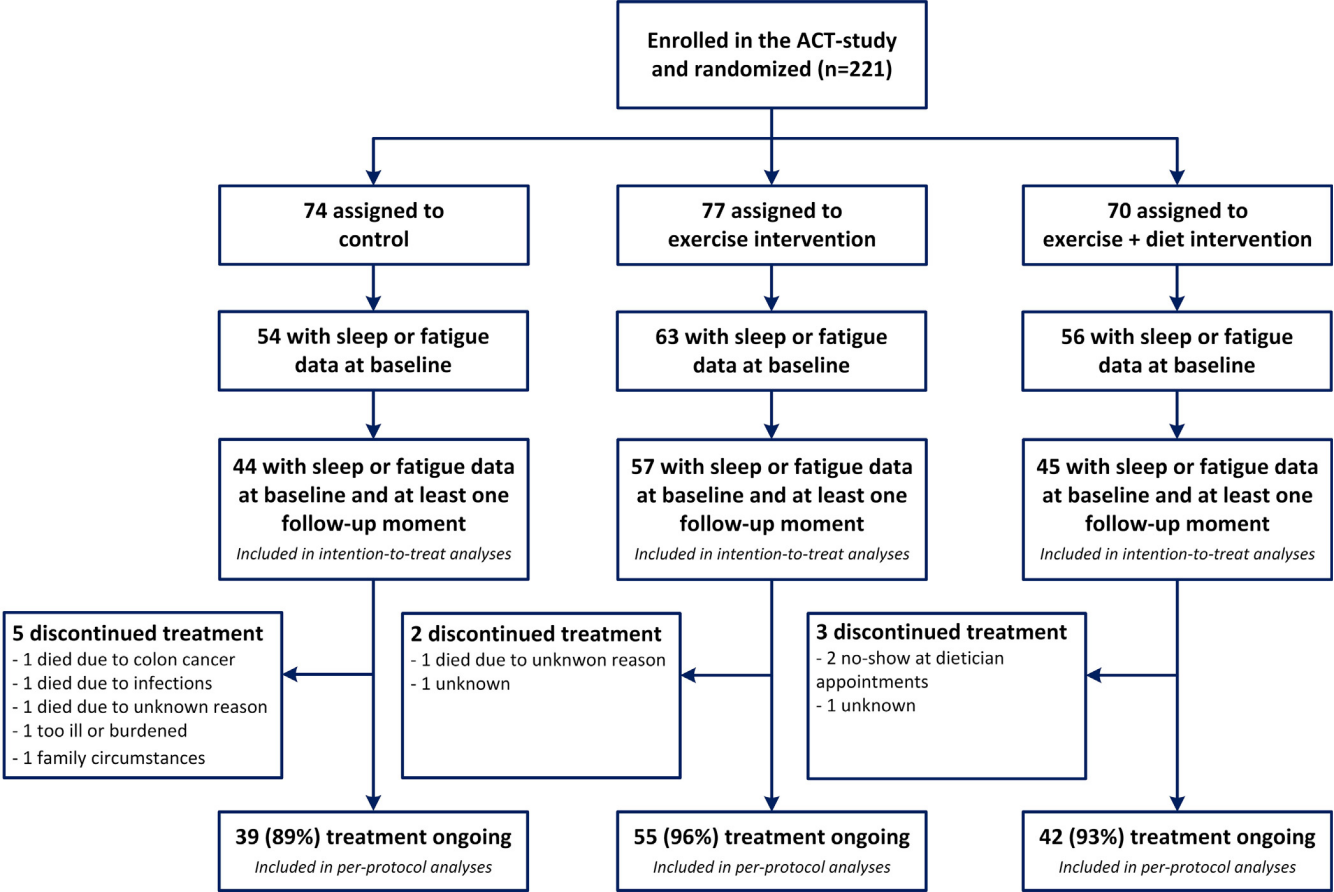
Participant flow diagram.

The flow of participants, including reasons for study discontinuation, is presented in Figure 2. We included 146 out of 221 KTRs. Compared to participants excluded in analyses, participants included in the analyses were older, had a longer time since transplantation, had less frequent dialysis pretransplantation or postmortal donors, were less frequently active smokers, and had higher systolic blood pressure (Supplementary Table S2). Among the included KTRs, 36% was female and mean age was 54 ± 13 years. Mean estimated glomerular filtration rate was 50 ± 15 ml/min per 1.73 m^2^, and median time posttransplantation was 6 (Interquartile range: 4–9) months. In total, 89% received triple immunosuppression. Forty-four were allocated to usual care, 57 to exercise intervention, and 44 to exercise plus diet intervention. Baseline characteristics were comparable across groups (Table 1). At baseline, mean sleep score was 53 ± 14 AU (range: 13–90) and mean fatigue severity score was 31 ± 11 AU (range: 8–56). Both sleep and fatigue severity scores were comparable across groups at baseline (*P* = 0.69 and *P* = 0.71, respectively). Among the 44 participants allocated to the control group, 5 discontinued (11%) (1 died because of colon cancer, 1 died because of infections, 1 died because of unknown reason, 1 got very ill, and 1 had family circumstances), and 39 (89%) completed the study. Among the 57 participants allocated to the exercise intervention group, 2 (4%) discontinued (1 died because of unknown reason, 1 unknown) and 55 (96%) completed the study. Among the 45 participants allocated to the exercise plus diet intervention group, 3 (7%) discontinued (2 because of no shows at dietician appointments, 1 unknown reason; none of the participants died) and 42 (93%) completed the study. In total, 96% of the participants with severe fatigue at baseline completed the study.

**Table 1:** Baseline characteristics

Variable	No intervention	Exercise intervention	Exercise + diet intervention
	*n* = 44	*n* = 57	*n* = 45
Female sex, *n* (%)	15 (34)	17 (30)	21 (47)
Time since transplantation, mos	7 [4, 9]	6 [4, 10]	6 [4, 7]
Age, yrs	54 ± 14	56 ± 12	52 ± 13
History of dialysis, *n* (%)	29 (66)	38 (67)	29 (64)
Donor type, n (%)			
Living donation	31 (71)	35 (61)	30 (67)
Donation after brain death	5 (11)	8 (14)	8 (18)
Donation after circulatory death	8 (18)	14 (25)	7 (16)
Body mass index, kg/m^2^	27 ± 5	26 ± 4	27 ± 5
Educational level, *n* (%)			
Low	16 (38)	26 (46)	11 (25)
Medium	15 (36)	18 (32)	20 (46)
High	11 (26)	12 (21)	13 (30)
Systolic blood pressure, mm Hg	140 ± 20	139 ± 18	138 ± 17
Diastolic blood pressure, mm Hg	80 ± 11	81 ± 11	78 ± 11
Active smoker, *n* (%)	2 (5)	4 (7)	6 (13)
History of smoking, *n* (%)	27 (66)	33 (60)	26 (58)
History of cardiovascular disease, *n* (%)	14 (32)	28 (49)	19 (42)
History of lung disease, *n* (%)	1 (2)	7 (12)	3 (7)
History of malignancy, *n* (%)	4 (9)	4 (7)	6 (13)
History of TIA or CVA, *n* (%)	2 (5)	4 (7)	2 (4)
History of diabetes, *n* (%)	13 (30)	20 (35)	15 (33)
History of hypertension, *n* (%)	27 (61)	32 (56)	25 (56)
Hemoglobin, g/dl	12.5 ± 1.7	12.7 ± 1.9	12.5 ± 1.6
Serum creatinine, mg/dl	131 [110–156]	135 [118–167]	127 [105–156]
eGFR, ml/min per 1.73m^2^	52 ± 15	48 ± 15	52 ± 17
Albumin, g/dl	4.3 ± 0.4	4.3 ± 0.4	4.4 ± 0.3
Triple immunosuppression, *n* (%)	37 (84)	49 (86)	43 (96)
Prednisolone use, *n* (%)	40 (91)	52 (91)	45 (100)
Calcineurin inhibitor use, *n* (%)	42 (96)	56 (98)	43 (96)
Proliferation inhibitor use, *n* (%)	40 (91)	53 (93)	40 (89)
mTor inhibitor use, *n* (%)	3 (7)	1 (2)	5 (11)

CVA, cardiovascular accident; eGFR, estimated glomerular filtration rate; mTOR, mammalian target of rapamycin; TIA, transient ischemic attack.

Normally distributed data were presented as mean ± SD, nonnormally data as median [interquartile range] and categorical data as number (valid %). Differences between groups were assessed using 1-way analysis of variance test, Kruskal Wallis test, or chi-square test, depending on the data distribution. Data regarding educational level, history of smoking, body mass index, and albumin were missing in 4 (3%), 4 (3%), 2 (1%), and 3 (2%) participants, respectively.

### Sleep Quality

**Figure 3:**
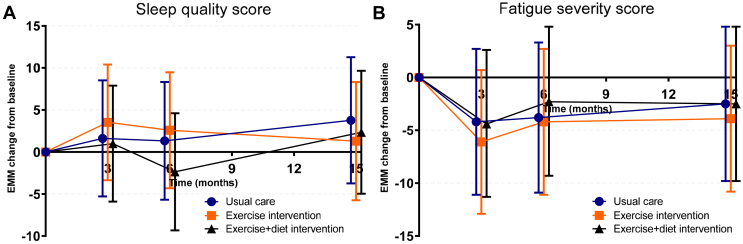
Trajectories of sleep quality and fatigue severity across groups. Higher scores indicate better sleep quality and greater fatigue severity. Changes are presented in change from baseline, showing estimated marginal means with their 95% confidence intervals derived from general linear mixed model analyses adjusted for study center.

**Figure 4:**
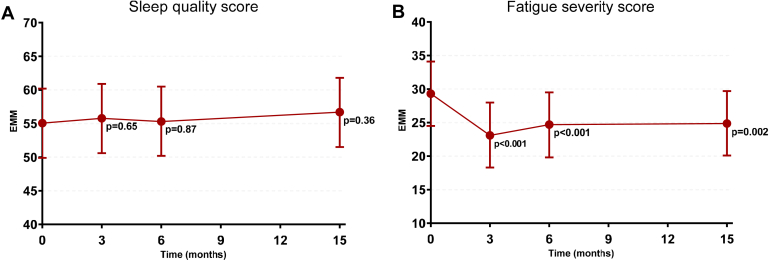
Trajectories of sleep quality and fatigue severity during the study period while combining all groups. Higher scores indicate better sleep quality and greater fatigue severity. *P*-values reflect the statistical significance of the difference compared to the baseline assessment. Estimated marginal means and their 95% confidence intervals were derived from general linear mixed model analyses adjusted for study center in analyses, in which all groups were combined, because no significant group differences were observed in other analyses.

The sleep quality trajectories across groups were generally comparable (intention-to-treat analyses, Figure 3a), with comparable mean differences from baseline at 3 months (i.e., end of the supervised exercise therapy) and 15 months (i.e., end of study) postbaseline. The mean difference from baseline at 15 months compared with the control group was −2.5 AU (95% CI: −9.1 to 4.2, *P* = 0.46) for the exercise group and −1.4 AU (95% CI: −8.4 to 5.6, *P* = 0.69) for the exercise plus diet group (a lower score reflects worse sleep quality) (Supplementary Table S3). Analysis in which the 3 groups were combined, did not show an improvement in sleep compared with baseline during the study (*P* > 0.36 at each time point) (Figure 4a).

### Fatigue Severity

Comparable fatigue severity trajectories were observed for all 3 groups (intention-to-treat analyses). Fatigue severity score decreased in all groups at 3 months postbaseline, whereas this decrease was less pronounced at 15 months postbaseline (Figure 3b). The between-group difference at 3 months postbaseline was −1.7 AU (95% CI: −5.8 to 2.4, *P* = 0.40) for the exercise intervention group and −0.2 AU (95% CI: −4.5 to 4.2, *P* = 0.95) for the exercise plus diet intervention group (lower score reflects less fatigue). In addition, at 15 months postbaseline, these differences were not statistically significant, with a between-group difference of −1.4 AU (95% CI: −7.0 to 4.2, *P* = 0.62) for the exercise intervention group and −0.2 AU (95% CI: −6.2 to 5.8, *P* = 0.94) for the exercise plus diet intervention group (Supplementary Table S3). However, analysis in which the 3 groups were combined showed that fatigue improved compared with baseline at each time point (*P* ≤ 0.001) (Figure 4b). Similarly, the prevalence of severe fatigue reduced from 32% at baseline to 21% at 15 months postbaseline.

### Effect Modifications

**Figure 5:**
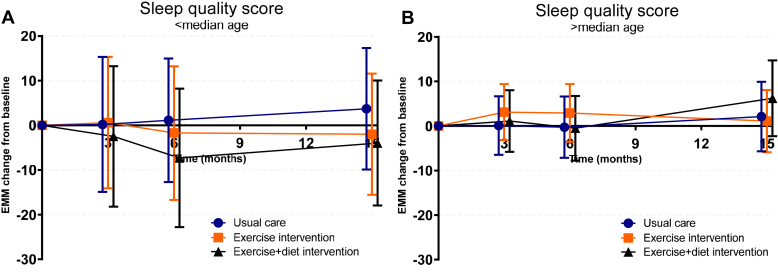
Trajectories of sleep quality across groups, stratified by age and baseline sleep quality score. Higher scores indicate better sleep quality. Age significantly affected the treatment effect on sleep. Changes are presented in change from baseline, showing estimated marginal means with their 95% confidence intervals derived from general linear mixed model analyses adjusted for study center.

A significant effect modification for sleep by age (exercise intervention: *P*_interaction_ = 0.11; exercise plus diet intervention: *P*_interaction_ = 0.031) was observed. The trajectories of sleep quality across groups, stratified by age, are presented in Figure 5. In both participants aged below and above the median age, neither intervention improved sleep quality at 15 months postbaseline. However, among participants aged below the median age, the intervention groups showed lower sleep scores (i.e., worse sleep quality) at 6 and 15 months postbaseline compared with the control, with a significant decrease in sleep score in the exercise plus diet group at 6 months postbaseline (−8.4 AU, 95% CI: −16.5 to −0.3, *P* = 0.043), as presented in Supplementary Table S4. No effect modifications on the treatment effect on sleep were identified for sex, time since transplantation, history of dialysis, donor type, educational level, systolic blood pressure, active smoker, or baseline severe fatigue or sleep quality; and none of the assessed variables were found to influence the treatment effect on fatigue severity.

### Per Protocol Analyses

In total, 10 participants (5 control, 2 exercise intervention, and 3 exercise plus diet intervention) did not adhere to or complete the allocated treatment and were excluded in the per-protocol analyses (Figure 2). Baseline characteristics of the patients included in these analyses are presented in Supplementary Table S5. Participants who did not adhere to or complete the allocated treatment had comparable sleep (49 ± 8 AU vs. 53 ± 14 AU, *P* = 0.33) and fatigue (28 ± 8 AU vs. 29 ± 12 AU, *P* = 0.89) scores. In addition, the results of the per-protocol analyses were comparable to the intention-to-treat analyses (Supplementary Table S6).

### Sensitivity Analyses

Analyses using absolute values instead of difference compared with baseline yielded comparable results for both sleep and fatigue, showing comparable trajectories and no significant between-group differences (Supplementary Figure S1).

## Discussion

Although exercise intervention has previously been shown to improve physical functioning,^13^ no treatment effect was observed on self-reported sleep or fatigue. Similarly, no treatment effect was found of the exercise plus diet intervention. We observed generally comparable sleep and fatigue trajectories across groups, and did not identify a subgroup in which the interventions had a beneficial effect on sleep or fatigue. However, fatigue did improve during the study compared with baseline in analyses combining the groups, whereas no improvement in sleep was observed. Still, 21% experienced severe fatigue at the end of the study. In addition, participants who dropped out during the study had comparable sleep and fatigue scores compared with those who completed the study.

Although the study was not powered to detect differences in sleep or fatigue, it appears unlikely that a larger sample size would have yielded clinically relevant different results. First, we observed highly comparable sleep and fatigue trajectories across groups. Second, the point estimates across groups at the end of the study were close to each other. For sleep, both intervention groups showed numerically lower scores at the end of the study, indicating worse sleep compared with the control group. However, these differences were not statistically significant and are unlikely clinically relevant, given the small effect sizes (Cohen’s *d* ranging from −0.18 to −0.10).^26^ Similarly, though the exercise intervention showed a numerically greater decrease in fatigue severity scores at all time points, indicating less fatigue compared with the other groups, this difference was small, with a Cohen’s *d* of −0.13 at the end of the study.^26^ Given that the thresholds for fatigue range significantly, with scores < 27 indicating no fatigue and those > 35 reflecting severe fatigue,^20^ the 1.4-unit change is minimal and falls well short of crossing any meaningful clinical threshold. The effect of the exercise plus diet intervention on fatigue was negligible (Cohen’s *d*: −0.02). Third, power calculations indicate that the current sample size of our study (*n* = 147) allowed for detection of between-group differences in sleep and fatigue of approximately 7.0 and 5.5 units, respectively. However, of the total of 221 participants randomized, some were excluded because of discontinuation or missing data on sleep or fatigue. Had all 221 participants completed the study and available sleep or fatigue data, we would have been able to detect differences of approximately 5.7 and 4.5 units for sleep and fatigue, respectively. To achieve statistical significance for the observed sleep differences (−2.5 units for the exercise intervention and −1.4 units for the exercise plus diet intervention), sample sizes of 1100 and 3353, respectively, would be required. For fatigue, using the observed fatigue differences (−1.4 units for the exercise intervention and −0.2 units for the exercise plus diet intervention), the required sample sizes would be 2284 and 106,845, respectively. These calculations further underscore the clinical irrelevance of the observed differences.

Although our findings seem to contrast with a small study among 26 liver transplant recipients and KTRs, which reported an improvement in fatigue after 12 months of supervised exercise therapy,^27^ this difference could be explained by the lack of control group in that study. In the current study, we observed an improvement in fatigue compared to baseline, but this improvement was seen across all groups, including the control group. To best of our knowledge, no other intervention study has assessed the effect of exercise or diet on sleep in KTRs.

Our findings differ with many intervention studies in other patient populations, such as individuals with chronic fatigue syndrome or cancer-induced fatigue, where exercise or dietary interventions did improve fatigue.^5^^,^^7^^,^^8^ In addition, studies in both healthy and clinical populations have demonstrated that nutrient therapy can alleviate fatigue.^28^, ^29^, ^30^ A Cochrane meta-analysis also concluded that exercise had probably an effect on fatigue in individuals with chronic kidney disease, although the evidence for its effect on sleep was deemed very uncertain.^31^ Another Cochrane meta-analysis among individuals with kidney failure requiring dialysis concluded that exercise improves fatigue compared to placebo or standard care.^9^ Multiple populations without sleep problems experienced improvements after dietary interventions,^6^ and middle-aged and older individuals with sleep complaints reported better sleep across various sleep domains after participating in an exercise training program.^4^ The discrepancy between our findings and those from other populations may stem from the unique etiology of sleep problems and fatigue in KTRs, which remains largely unraveled but appears to differ from that in other populations. For example, though proton pump inhibitors have not been linked with fatigue in other populations, they have been associated with fatigue in KTRs.^21^ Furthermore, psychological and social factors, such as behavior in response to fatigue, might play a more significant role than in KTRs compared with other populations.^32^ Future research should aim to unravel the underlying causes of poor sleep quality and fatigue in KTRs, because these symptoms are highly prevalent. Sleep problems affect approximately 33% of men and 49% of women,^2^ and fatigue impacts 40% to 50% of KTRs.^3^

Another possible explanation for the lack of treatment effects on fatigue in this study could be that the increased energy levels allowed patients to participate in more daily activities, potentially leading to activity-induced fatigue. Certainly, they participated in an intense 2-weekly exercise program in the first 3 months. Notably, almost all participants with severe fatigue at baseline were able to adhere to the study protocol or complete the study, and patients who dropped out were comparable in sleep and fatigue scores at baseline. Therefore, we can conclude that participation in this program was feasible despite (severe) fatigue, and did not aggravate the fatigue. Moreover, the subjective measurement of both sleep and fatigue may have influenced our results, meaning improvements could have occurred but were not detected. However, self-reported data is valuable for capturing how patients experience their sleep quality and fatigue severity, emphasizing the importance of our findings from the patient’s perspective. Nevertheless, future studies could consider incorporating objective measures to more accurately assess these outcomes. Finally, the presence of sleep disorders may have influenced the treatment effects on sleep quality.

A key strength of this study is its randomized controlled design, which enhances both the applicability and validity of the findings. However, a portion of the ACT study participants were excluded because of missing questionnaire data, thereby potentially introducing a selection bias, with for example an underrepresentation of older patients. Although age affected the treatment effect, with subgroup analyses showing no significant effects among older patients, the difference in response to the exercise plus diet intervention may still have negatively impacted the overall group’s response, leading to higher point estimates for the effect of the exercise plus diet intervention on sleep. Nevertheless, this would likely not have altered the conclusions. Other differences between included and excluded participants did not affect sleep or fatigue outcomes. The missing sleep and fatigue data resulted in an imbalanced sample size across groups, which may limit comparison of the statistical significance, although group sizes were balanced at time of randomization. In addition, a participation bias may have occurred, because highly motivated individuals may have been more willing to participate in an exercise or diet studies because of the intensity of the interventions. In addition, individuals with sleep problems or fatigue may have lacked the energy or motivation to join a clinical trial like this one. However, the fatigue score in our study generally aligns with those from a Dutch kidney transplantation cohort (31 ± 11 AU vs. 29 ± 13 AU),^23^ and our participants exhibited worse sleep compared with another Dutch kidney transplantation cohort (53 ± 14 AU vs. 61 ± 20 AU).^16^ It can be considered a limitation of our study that there is a difference in recall periods between the KDQOL-SF and CIS20R instruments (4 vs. 2 weeks, respectively).In addition, sleep was assessed using the KDQOL-SF questionnaire. Although validated to assess HRQoL in the kidney transplant population, this questionnaire has not specifically been designed for detailed sleep assessment. More comprehensive sleep questionnaires, such as the Pittsburgh Sleep Quality Index, may provide additional insights and could be considered in future research.

Furthermore, because the diet intervention consisted of personalized dietary counseling without strict dietary rules for participants to follow, such as caloric restriction or intake of a certain amount of proteins per day, our study does not allow us to draw conclusions about the impact of specific dietary changes on the outcomes. This aspect remains to be investigated and may explain the different findings compared with other populations regarding the dietary intervention effect. However, the diet intervention in our study reflects real-world practice, where dietitians aim to adjust the diet by providing (personalized) counseling, rather forcing strict diet changes after kidney transplantation.

In conclusion, although patient-reported fatigue improves in the first year posttransplantation, (severe) fatigue remains highly prevalent and persists in KTRs. Participation in a lifestyle rehabilitation program after kidney transplantation is also feasible for KTRs with severe fatigue; however, it neither improves nor worsens the subjective experience of sleep or fatigue. Improvement of lifestyle, important as it may be, is not a panacea for KTRs. Our findings suggest that factors beyond lifestyle play a more significant role in the etiology of sleep and fatigue in KTRs, and that addressing sleep and fatigue in this population likely requires a specific, dedicated approach.

## Appendix

### List of ACTx Collaborators

Tim J. Knobbe, Daan Kremer, Dorien M. Zelle, Gerald Klaassen, Desie Dijkema, Iris M.Y. van Vliet, Paul B. Leurs, Frederike J. Bemelman, Maarten H.L. Christiaans, Stefan P. Berger, Gerjan Navis, Stephan J.L. Bakker, and Eva Corpeleijn

## Disclosure

All the authors declared no conflicting interests.

## Supplementary Material

Supplementary File (PDF) **Figure 1.** Trajectories of sleep quality and fatigue severity across groups while using absolute values instead of difference compared to baseline. **Table S1.** CONSORT 2010 checklist of information to include when reporting a randomized trial. **Table S2.** Characteristics of participants included and excluded in analyses. **Table S3.** Effect on sleep quality and fatigue severity. **Table S4.** Statistically significant effect modifications for sleep. **Table S5.** Characteristics of participants included and excluded in per-protocol analyses. **Table S6.** Per-protocol analyses: effect on sleep and fatigue.

## Footnotes

Supplementary File (PDF)
**Figure 1.** Trajectories of sleep quality and fatigue severity across groups while using absolute values instead of difference compared to baseline. **Table S1.** CONSORT 2010 checklist of information to include when reporting a randomized trial. **Table S2.** Characteristics of participants included and excluded in analyses. **Table S3.** Effect on sleep quality and fatigue severity. **Table S4.** Statistically significant effect modifications for sleep. **Table S5.** Characteristics of participants included and excluded in per-protocol analyses. **Table S6.** Per-protocol analyses: effect on sleep and fatigue.

## References

[1] Wang Y., Hemmelder M.H., Bos W.J.W. (2021). Mapping health-related quality of life after kidney transplantation by group comparisons: a systematic review. Nephrol Dial Transplant.

[2] Knobbe T.J., Kremer D., Eisenga M.F. (2023). Sleep quality, fatigue, societal participation and health-related quality of life in kidney transplant recipients: a cross-sectional and longitudinal cohort study. Nephrol Dial Transplant.

[3] Bossola M., Arena M., Urciuolo F. (2021). Fatigue in kidney transplantation: a systematic review and meta-analysis. Diagnostics (Basel).

[4] Yang P.Y., Ho K.H., Chen H.C., Chien M.Y. (2012). Exercise training improves sleep quality in middle-aged and older adults with sleep problems: a systematic review. J Physiother.

[5] Larun L., Brurberg K.G., Odgaard-Jensen J., Price J.R. (2019). Exercise therapy for chronic fatigue syndrome. Cochrane Database Syst Rev.

[6] Godos J., Grosso G., Castellano S., Galvano F., Caraci F., Ferri R. (2021). Association between diet and sleep quality: a systematic review. Sleep Med Rev.

[7] Barnish M., Sheikh M., Scholey A. (2023). Nutrient therapy for the improvement of fatigue symptoms. Nutrients.

[8] Tomlinson D., Diorio C., Beyene J., Sung L. (2014). Effect of exercise on cancer-related fatigue: a meta-analysis. Am J Phys Med Rehabil.

[9] Natale P., Ju A., Strippoli G.F.M. (2023). Interventions for fatigue in people with kidney failure requiring dialysis. Cochrane Database Syst Rev.

[10] Gomes Neto A.W., Boslooper-meulenbelt K., Geelink M. (2020). Protein intake, fatigue and quality of life in stable outpatient kidney transplant recipients. Nutrients.

[11] Ujszaszi A., Czira M.E., Fornadi K., Novak M., Mucsi I., Molnar M.Z. (2012). Quality of life and protein-energy wasting in kidney transplant recipients. Int Urol Nephrol.

[12] Rodrigue J.R., Mandelbrot D.A., Hanto D.W., Johnson S.R., Karp S.J., Pavlakis M. (2011). A cross-sectional study of fatigue and sleep quality before and after kidney transplantation. Clin Transpl.

[13] Knobbe T.J., Kremer D., Zelle D.M. (2024). Effect of an exercise intervention or combined exercise and diet intervention on health-related quality of life-physical functioning after kidney transplantation: the Active Care after Transplantation (ACT) multicentre randomised controlled trial. Lancet Healthy Longev.

[14] Schulz K.F., Altman D.G., Moher D., CONSORT Group (2010). IGF-I induced genes in stromal fibroblasts predict the clinical outcome of breast and lung cancer patients. BMC Med.

[15] Klaassen G., Zelle D.M., Navis G.J. (2017). Lifestyle intervention to improve quality of life and prevent weight gain after renal transplantation: design of the Active Care after Transplantation (ACT) randomized controlled trial. BMC Nephrol.

[16] Barotfi S., Molnar M.Z., Almasi C. (2006). Validation of the Kidney Disease Quality of Life-Short Form questionnaire in kidney transplant patients. J Psychosom Res.

[17] Hays R., Kallich J., Mapes D., Coons S., Carter W. (1994). Development of the kidney disease quality of life (KDQOL) instrument. Qual Life Res.

[18] Beurskens A.J.H.M., Bültmann U., Ij K., Vercoulen J.H.M.M., Bleijenberg G., Swaen G.M.H. (2000). Fatigue among working people: validity of a questionnaire measure. Occup Environ Med.

[19] Vercoulen J.H.M.M., Swanink C.M., Fennis J.F., Galama J.M., van der Meer J.W., Bleijenberg G. (1994). Dimensional assessment of chronic fatigue syndrome. J Psychosom Res.

[20] Worm-Smeitink M., Gielissen M., Bloot L. (2017). The assessment of fatigue: psychometric qualities and norms for the Checklist individual strength. J Psychosom Res.

[21] Knobbe T.J., Kremer D., Douwes R.M. (2023). Proton pump inhibitor use, fatigue, and health-related quality of life in kidney transplant recipients: results from the TransplantLines biobank and cohort study. Am J Kidney Dis.

[22] Kremer D., Knobbe T.J., Vinke J.S.J. (2024). Iron deficiency, anemia, and patient-reported outcomes in kidney transplant recipients. Am J Transplant.

[23] Knobbe T.J., Kremer D., Eisenga M.F. (2021). Airflow limitation, fatigue, and health-related quality of life in kidney transplant recipients. Clin J Am Soc Nephrol.

[24] Knobbe T.J., Kremer D., Eisenga M.F. (2022). Hand dexterity, daily functioning and health-related quality of life in kidney transplant recipients. Sci Rep.

[25] Inker L.A., Eneanya N.D., Coresh J. (2021). New creatinine- and cystatin C–based equations to estimate GFR without race. N Engl J Med.

[26] Sullivan G.M., Feinn R. (2012). Using effect size—or why the P value is not enough. J Grad Med Educ.

[27] Roi G.S., Stefoni S., Mosconi G. (2014). Physical activity in solid organ transplant recipients: organizational aspects and preliminary results of the Italian project. Transplant Proc.

[28] Saito H., Cherasse Y., Suzuki R., Mitarai M., Ueda F., Urade Y. (2017). Zinc-rich oysters as well as zinc-yeast- and astaxanthin-enriched food improved sleep efficiency and sleep onset in a randomized controlled trial of healthy individuals. Mol Nutr Food Res.

[29] Hansen A.L., Dahl L., Olson G. (2014). Fish consumption, sleep, daily functioning, and heart rate variability. J Clin Sleep Med.

[30] Afaghi A., O’Connor H., Chow C.M. (2007). High-glycemic-index carbohydrate meals shorten sleep onset. Am J Clin Nutr.

[31] Natale P., Ruospo M., Saglimbene V.M., Palmer S.C., Strippoli G.F. (2019). Interventions for improving sleep quality in people with chronic kidney disease. Cochrane Database Syst Rev.

[32] Sands I., Picariello F., Maple H., Chilcot J. (2022). Psychosocial and clinical associations of fatigue severity and fatigue-related impairment in kidney transplant recipients. Behav Med.

