# Supplementary material for: Effect of Exercise or Combined Exercise and Diet Intervention on Sleep and Fatigue in Kidney Transplant Recipients
Source: Kidney Int Rep. 2025 Apr 10;10(7):2233–42. doi: 10.1016/j.ekir.2025.04.008 (PMC12266161; doi:10.1016/j.ekir.2025.04.008)
Supplement: Supplementary File (PDF) — Figure 1. Trajectories of sleep quality and fatigue severity across groups while using absolute values instead of difference compared to baseline. Table S1. CONSORT 2010 checklist of information to include when reporting a randomized trial. Table S2. Characteristics of participants included and excluded in analyses. Table S3. Effect on sleep quality and fatigue severity. Table S4. Statistically significant effect modifications for sleep. Table S5. Characteristics of participants included and excluded in per-protocol analyses. Table S6. Per-protocol analyses: effect on sleep and fatigue. [file mmc1.pdf]

Supplementary material

Effect of Exercise Intervention or Combined Exercise and Diet Intervention on Self-Reported Sleep and Fatigue in Kidney Transplant Recipients: A Predefined Secondary Analysis of the Active Care after Transplantation (ACT) Randomized Clinical Trial

Table of content

Supplemental Table 1. CONSORT 2010 checklist of information to include when reporting a randomised trial. 2
Supplemental Table 2. Characteristics of participants included and excluded in analyses. 5
Supplemental Table 3. Effect on sleep quality and fatigue severity. 6
Supplemental Table 4. Statistically significant effect modifications for sleep. 7
Supplemental Table 5. Characteristics of participants included and excluded in per-protocol analyses. 8
Supplemental Table 6. Per-protocol analyses: effect on sleep and fatigue. 9
Supplemental Figure 1. Trajectories of sleep quality and fatigue severity across groups while using absolute values instead of difference compared to baseline. 10

Supplemental Table 1. CONSORT 2010 checklist of information to include when reporting a randomised trial.

Section/Topic	Item No	Checklist item	Reported
Title and abstract			
	1a	Identification as a randomised trial in the title	title
	1b	Structured summary of trial design, methods, results, and conclusions (for specific guidance see CONSORT for abstracts)	Abstract
Introduction			
Background and objectives	2a	Scientific background and explanation of rationale	Intro par 1-2
	2b	Specific objectives or hypotheses	Intro par 3
Methods			
Trial design	3a	Description of trial design (such as parallel, factorial) including allocation ratio	Meth par 1,3
	3b	Important changes to methods after trial commencement (such as eligibility criteria), with reasons	Primary publication*
Participants	4a	Eligibility criteria for participants	Meth par 2, primary publication*
	4b	Settings and locations where the data were collected	Meth par 2
Interventions	5	The interventions for each group with sufficient details to allow replication, including how and when they were actually administered	Meth par 5-6, primary publication* including study protocol
Outcomes	6a	Completely defined pre-specified primary and secondary outcome measures, including how and when they were assessed	This concerned a pre-defined secondary outcome, Meth par 7
	6b	Any changes to trial outcomes after the trial commenced, with reasons	primary publication*
Sample size	7a	How sample size was determined	Meth par 2, Fig 2
	7b	When applicable, explanation of any interim analyses and stopping guidelines	n/a

Supplemental Table 1. (continued)

Section/Topic	Item No	Checklist item	Reported
Randomisation:			
Sequence generation	8a	Method used to generate the random allocation sequence	Meth par 3
	8b	Type of randomisation; details of any restriction (such as blocking and block size)	Meth par 3
Allocation concealment mechanism	9	Mechanism used to implement the random allocation sequence (such as sequentially numbered containers), describing any steps taken to conceal the sequence until interventions were assigned	Meth par 3
Implementation	10	Who generated the random allocation sequence, who enrolled participants, and who assigned participants to interventions	Meth par 3, primary publication*
Blinding	11a	If done, who was blinded after assignment to interventions (for example, participants, care providers, those assessing outcomes) and how	n/a
	11b	If relevant, description of the similarity of interventions	n/a
Statistical methods	12a	Statistical methods used to compare groups for primary and secondary outcomes	Meth par 10
	12b	Methods for additional analyses, such as subgroup analyses and adjusted analyses	Meth par 11-12
Results			
Participant flow (a diagram is strongly recommended)	13a	For each group, the numbers of participants who were randomly assigned, received intended treatment, and were analysed for the primary outcome	Res par 1, Fig 2
	13b	For each group, losses and exclusions after randomisation, together with reasons	Fig 2, Res par 5
Recruitment	14a	Dates defining the periods of recruitment and follow-up	Meth par 1, primary publication*
	14b	Why the trial ended or was stopped	Primary publication*
Baseline data	15	A table showing baseline demographic and clinical characteristics for each group	Table 1
Numbers analysed	16	For each group, number of participants (denominator) included in each analysis and whether the analysis was by original assigned groups	Supp.Table 3,4,6
Outcomes and estimation	17a	For each primary and secondary outcome, results for each group, and the estimated effect size and its precision (such as 95% confidence interval)	Supp.Table 3,4,6
	17b	For binary outcomes, presentation of both absolute and relative effect sizes is recommended	n/a
Ancillary analyses	18	Results of any other analyses performed, including subgroup analyses and adjusted analyses, distinguishing pre-specified from exploratory	Supp.Table 4,6

Supplemental Table 1. (continued)

Section/Topic	Item No	Checklist item	Reported
Ancillary analyses	18	Results of any other analyses performed, including subgroup analyses and adjusted analyses, distinguishing pre-specified from exploratory	Supp.Table 4,6
Harms	19	All important harms or unintended effects in each group (for specific guidance see CONSORT for harms)	Primary publication*
Discussion			
Limitations	20	Trial limitations, addressing sources of potential bias, imprecision, and, if relevant, multiplicity of analyses	Discussion par 2,5,6
Generalisability	21	Generalisability (external validity, applicability) of the trial findings	Discussion par 6
Interpretation	22	Interpretation consistent with results, balancing benefits and harms, and considering other relevant evidence	Discussion par 1-7
Other information			
Registration	23	Registration number and name of trial registry	Meth par 1, abstract
Protocol	24	Where the full trial protocol can be accessed, if available	Primary publication, referenced multiple times
Funding	25	Sources of funding and other support (such as supply of drugs), role of funders	Funding

Citation: Schulz KF, Altman DG, Moher D, for the CONSORT Group. CONSORT 2010 Statement: updated guidelines for reporting parallel group randomised trials. BMC Medicine. 2010;8:18.

© 2010 Schulz et al. This is an Open Access article distributed under the terms of the Creative Commons Attribution License (http://creativecommons.org/licenses/by/2.0), which permits unrestricted use, distribution, and reproduction in any medium, provided the original work is properly cited.

Abbreviations: disc, discussion; intro, introduction; meth, methods; res, results; S.Table, Supplemental Table

*: Knobbe TJ, Kremer D, Zelle DM, Klaassen G, Dijkema D, van Vliet IMY, Leurs PB, Bemelman FJ, Christiaans MHL, Berger SP, Navis G, Bakker SJL, Corpeleijn E. Effect of an exercise intervention or combined exercise and diet intervention on health-related quality of life-physical functioning after kidney transplantation: the Active Care after Transplantation (ACT) multicentre randomised controlled trial. Lancet Healthy Longev. 2024 Sep;5(9):100622. doi: 10.1016/j.lanhl.2024.07.005. Epub 2024 Sep 10. PMID: 39270688.

Supplemental Table 2. Characteristics of participants included and excluded in analyses.

Variable	Excluded in analyses N=75	Included in analyses N=146	P-value
Female sex, *n* (%)	30 (40)	53 (36.3)	0.70
Time since transplantation, months	5 [3, 8]	6 [4, 9]	0.037
Age, years	49 (14)	54 (13)	0.009
History of dialysis, *n* (%)	62 (83)	96 (66)	0.013
Donor type, n (%)			0.002
Living donation	31 (41)	96 (66)	
Donation after brain death	20 (27)	21 (14)	
Donation after circulatory death	24 (32)	29 (20)	
Body mass index, kg/m^2	28 ± 5	27 ± 5	0.19
Educational level, *n* (%)			0.30
Low	12 (46)	53 (37)	
Medium	11 (42)	53 (37)	
High	3 (12)	36 (25)	
Systolic blood pressure, mmHg	134 ± 15	139 ± 18	0.029
Diastolic blood pressure, mmHg	78 ± 11	80 ± 11	0.33
Active smoker, *n* (%)	14 (22)	12 (8)	0.011
History of smoking, *n* (%)	36 (63)	86 (61)	0.90
History of cardiovascular disease, *n* (%)	28 (37)	61 (42)	0.62
History of lung disease, *n* (%)	10 (13)	11 (8)	0.25
History of malignancy, *n* (%)	6 (8)	14 (10)	0.89
History of TIA or CVA, *n* (%)	5 (7)	8 (6)	0.96
History of diabetes, *n* (%)	25 (33)	48 (33)	1.00
History of hypertension, *n* (%)	49 (65)	84 ± 58	0.33
Hemoglobin, g/dL	12.3 ± 2.1	12.6 ± 1.76	0.21
Serum creatinine, mg/dL	135 [120, 169]	134 [111, 161]	0.34
eGFR, $ml/min/1.73m^2$	50 ± 19	50 ± 15	0.88
Albumin, g/dL	4.4 ± 0.3	4.3 ± 0.4	0.14
Triple immunosuppression, *n* (%)	67 (89)	129 (88)	1.00
Prednisolon use, *n* (%)	68 (91)	137 (94)	0.56
Calcineurin inhibitor use , *n* (%)	74 (99)	141 (97)	0.64
Proliferation inhibitor use, *n* (%)	73 (97)	133 (91)	0.14
mTor inhibitor use, *n* (%)	2 (3)	9 (6)	0.42

Normally distributed data were presented as mean ± SD, non-normally data as median [interquartile range] and categorical data as number (valid %). Differences between groups were assessed using independent T-test, Mann-Whitney U test or chi-square test, depending on the data distribution. Data regarding educational level, active smoking, history of smoking, body mass index and albumin were missing in 53 (24%), 11 (5%), 23 (10%), 3 (1%) and 3 (1%) participants, respectively. Abbreviations: CVA, cardiovascular accident; eGFR, estimated glomerular filtration rate; mTOR, mammalian target of rapamycin; TIA, transient ischemic attack.

Supplemental Table 3. Effect on sleep quality and fatigue severity.

		Control	Exercise intervention			Exercise + diet intervention		
	df	Mean	Mean	Difference to control	P-value	mean	Difference to control	P-value
**Sleep quality**								
3 months post-baseline	132	+1.6<br>(-5.3, +8.5)	+3.5<br>(-3.4, +10.4)	+1.9<br>(-2.5, +6.3)	0.39	1.0<br>(-5.9, +7.9)	-0.6<br>(-5.3, +4.1)	0.79
6 months post-baseline	126	+1.3<br>(-5.7, +8.3)	+2.6<br>(-4.3, +9.5)	+1.3<br>(-3.8, +6.4)	0.63	-2.4<br>(-9.3, +4.6)	-3.7<br>(-9.1, +1.7)	0.18
15 months post baseline	134	+3.8<br>(-3.7, +11.3)	+1.3<br>(-5.7, +8.3)	-2.5<br>(-9.1, +4.2)	0.46	+2.3<br>(-5.0, +9.6)	-1.4<br>(-8.4, +5.6)	0.69
**Fatigue severity**								
3 months post-baseline	132	-4.2<br>(-11.1, +2.7)	-6.1<br>(-12.9, +0.7)	-1.9<br>(-6.0, +2.2)	0.36	-4.4<br>(-11.3, +2.6)	-0.2<br>(-4.5, +4.2)	0.95
6 months post-baseline	124	-3.8<br>(-10.9, +3.3)	-4.2<br>(-11.1, +2.7)	-0.4<br>(-5.6, +4.7)	0.87	-2.3<br>(-9.3, +4.8)	1.6<br>(-3.9, +7.0)	0.57
15 months post baseline	106	-2.5<br>(-9.8, +4.8)	-3.9<br>(-10.8, +3.0)	-1.4<br>(-7.0, +4.2)	0.62	-2.7<br>(-9.9, +4.5)	-0.2<br>(-6.2, +5.8)	0.94

Changes are presented in change from baseline, showing estimated marginal means (EMM) with their 95% confidence intervals derived from general linear mixed model analyses adjusted for study center. Abbreviation: AU, arbitrary unit.

Supplemental Table 4. Statistically significant effect modifications for sleep.

	df	Exercise intervention		Exercise + diet intervention	
		Difference to control	P-value	Difference to control	P-value
**Age <median of 54.7**					
3 months post-baseline	57	+0.4 (-7.1, +8.0)	0.91	-2.6 (-9.6, +4.3)	0.44
6 months post-baseline	49	-2.9 (-11.3, +5.6)	0.50	-8.4 (-16.5, -0.3)	0.043
15 months post baseline	53	-5.7 (-17.0, +5.6)	0.32	-7.7 (-18.5, +3.1)	0.16
**Age >median of 54.7**					
3 months post-baseline	71	+3.0 (-2.7, +8.8)	0.29	+1.0 (-5.7, +7.8)	0.76
6 months post-baseline	73	+3.2 (-3.4, +9.8)	0.34	-0.3 (-7.9, +7.3	0.94
15 months post baseline	76	-1.01 (-9.3, +7.3)	0.81	+4.1 (-5.6, +13.9)	0.40

Changes are presented in change from baseline, showing estimated marginal means (EMM) with their 95% confidence intervals derived from general linear mixed model analyses adjusted for study center. Abbreviation: AU, arbitrary unit. The following potential effect modifications were assessed: sex, age, time after transplantation, history of dialysis, educational level, severe fatigue at baseline or lower sleep quality at baseline (using the median value as the cut-off, due to lack of generally accepted standard).

Supplemental Table 5. Characteristics of participants included and excluded in per-protocol analyses.

Variable	Excluded in analyses N=10	Included in analyses N=136	P-value
Female sex, *n* (%)	0 (0)	53 (39.0)	0.033
Time since transplantation, months	7 [4, 9]	6 [4, 9]	0.85
Age, years	63 (12)	54 (13)	0.022
History of dialysis, *n* (%)	7 (70)	89 (65)	1.00
Donor type, n (%)			0.69
Living donation	6 (60)	90 (66)	
Donation after brain death	1 (10)	20 (15)	
Donation after circulatory death	3 (30)	26 (19)	
Body mass index, kg/m^2	29 ± 7	27 ± 4	0.14
Educational level, *n* (%)			0.88
Low	4 (40)	49 (37)	
Medium	3 (30)	50 (38)	
High	3 (30)	33 (25)	
Systolic blood pressure, mmHg	149 ± 25	138 ± 17	0.064
Diastolic blood pressure, mmHg	83 ± 11	80 ± 11	0.33
Active smoker, *n* (%)	2 (20)	10 (7)	0.42
History of smoking, *n* (%)	6 (67)	80 (61)	0.99
History of cardiovascular disease, *n* (%)	6 (60)	55 (40)	0.38
History of lung disease, *n* (%)	0 (0)	11 (8)	0.75
History of malignancy, *n* (%)	2 (20)	12 (9)	0.55
History of TIA or CVA, *n* (%)	0 (0)	8 (6)	0.95
History of diabetes, *n* (%)	3 (30)	45 (33)	1.00
History of hypertension, *n* (%)	6 (60)	78 (57)	1.00
Hemoglobin, g/dL	13.4 ± 1.5	12.5 ± 1.8	0.13
Serum creatinine, mg/dL	152 [104, 183]	132 [114, 160]	0.57
eGFR, ml/min/1.73m^2	52 ± 20	50 ± 15	0.71
Albumin, g/dL	4.2 ± 0.5	4.3 ± 0.3	0.50
Triple immunosuppression, *n* (%)	8 (80)	121 (89)	0.73
Prednisolon use, *n* (%)	8 (80)	129 (95)	0.23
Calcineurin inhibitor use , *n* (%)	10 (100)	131 (96)	1.00
Proliferation inhibitor use, *n* (%)	9 (90)	124 (91)	1.00
mTor inhibitor use, *n* (%)	1 (10)	8 (6)	1.00

Normally distributed data were presented as mean ± SD, non-normally data as median [interquartile range] and categorical data as number (valid %). Differences between groups were assessed using independent T-test, Mann-Whitney U test or chi-square test, depending on the data distribution. Data regarding educational level, history of smoking, body mass index and albumin were missing in 4 (3%), 5 (3%), 2 (1%) and 3 (2%) participants, respectively. Abbreviations: CVA, cardiovascular accident; eGFR, estimated glomerular filtration rate; mTOR, mammalian target of rapamycin; TIA, transient ischemic attack.

Supplemental Table 6. Per-protocol analyses: effect on sleep and fatigue.

		Control	Exercise intervention			Exercise + diet intervention		
	df	Mean	Mean	Difference to control	P-value	mean	Difference to control	P-value
**Sleep**								
3 months post-baseline	124	+1.3 (-6.1, +8.7)	+3.7 (-3.7, +11.0)	+2.4 (-2.3, +7.1)	0.31	+1.1 (-6.3, +8.5)	-0.2 (-5.2, +4.8)	0.94
6 months post-baseline	119	+1.6 (-5.9, +9.1)	+2.3 (-5.1, +9.7)	+0.7 (-4.6, +6.1)	0.79	-2.5 (-10.0, +4.9)	-4.1 (-9.7, +1.5)	0.15
15 months post baseline	127	+1.7 (-6.0, +9.5)	+1.1 (-6.3, +8.5)	-0.6 (-6.9, +5.6)	0.84	+0.1 (-7.5, +7.8)	-1.6 (-8.3, +5.0)	0.63
**Fatigue severity**								
3 months post-baseline	123	-4.5 (-11.4, 2.3)	-6.4 (-13.1, +0.4)	-1.8 (-6.1, +2.4)	0.40	-5.1 (-11.9, +1.8)	-0.5 (-5.1, +4.1)	0.82
6 months post-baseline	116	-3.8 (-10.8, +3.3)	-4.6 (-11.4, +2.2)	-0.8 (-6.1, +4.4)	0.76	-3.3 (-10.3, +3.7)	+0.5 (-5.1, +6.1)	0.86
15 months post baseline	105	-2.5 (-9.7, +4.7)	-4.1 (-10.9, 2.7)	-1.6 (-7.3, +4.0)	0.57	-2.7 (-9.8, +4.4)	-0.2 (-6.3, +5.8)	0.94

Changes are presented in change from baseline, showing estimated marginal means (EMM) with their 95% confidence intervals derived from general linear mixed model analyses adjusted for study center. Abbreviation: AU, arbitrary unit.

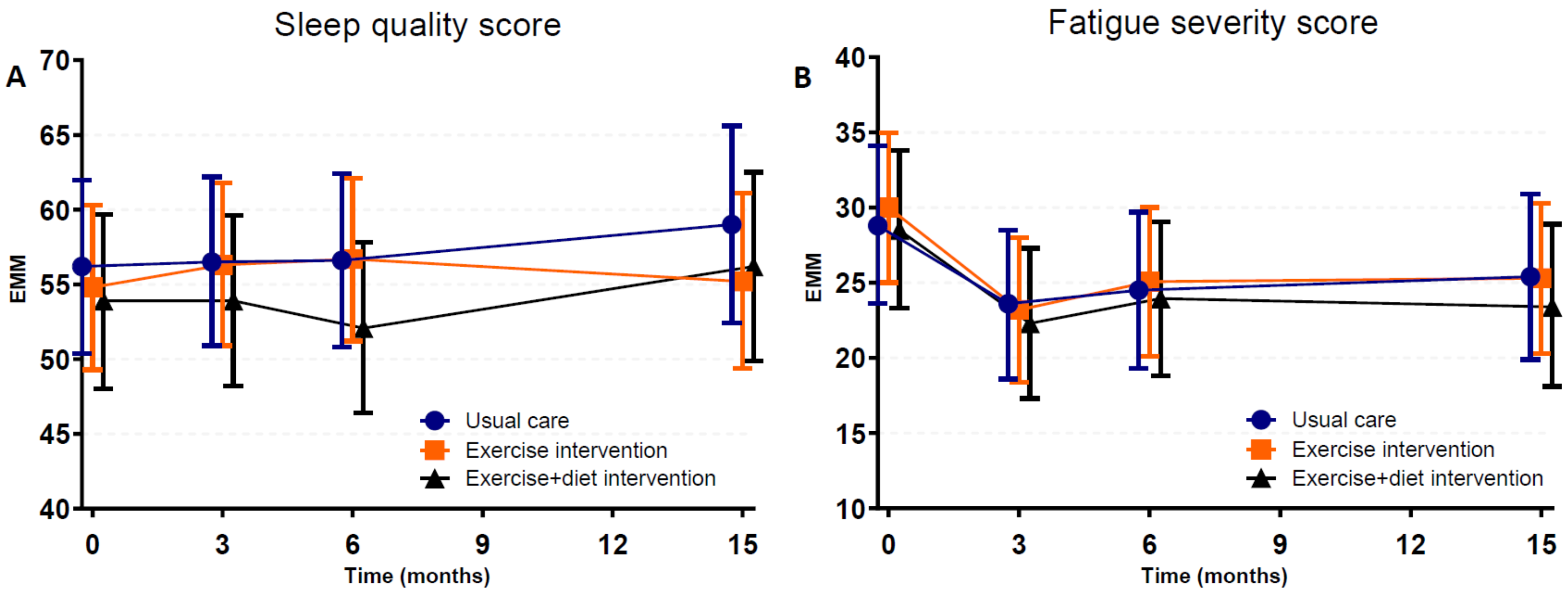

Supplemental Figure 1. Trajectories of sleep quality and fatigue severity across groups while using absolute values instead of difference compared to baseline. Higher scores indicate better sleep quality and greater fatigue severity. Estimated marginal means (EMM) and their 95% confidence intervals were derived from general linear mixed model analyses adjusted for study center.
